# Detecting and Localizing Cyber-Physical Attacks in Water Distribution Systems without Records of Labeled Attacks

**DOI:** 10.3390/s22166035

**Published:** 2022-08-12

**Authors:** Mashor Housh, Noy Kadosh, Jack Haddad

**Affiliations:** 1 Department of Natural Resources and Environmental Management, University of Haifa, Haifa 3498838, Israel; 2 Faculty of Civil and Environmental Engineering, Technion–Israel Institute of Technology, Haifa 3200003, Israel

**Keywords:** water cyber-physical systems, smart water systems, cyber security of water systems

## Abstract

Modern water distribution systems (WDSs) offer automated controls and operations to improve their efficiency and reliability. Nonetheless, such automation can be vulnerable to cyber-attacks. Therefore, various approaches have been suggested to detect cyber-attacks in WDSs. However, most of these approaches rely on labeled attack records which are rarely available in real-world applications. Thus, for a detection model to be practical, it should be able to detect and localize events without referring to a predetermined list of labeled attacks. This study proposes a semi-supervised approach that relies solely on attack-free datasets to address this challenge. The approach utilizes a reduction in dimensionality by using maximum canonical correlation analysis (MCCA) followed by support vector data description (SVDD). The developed algorithm was tested on two case studies and various datasets, demonstrating consistently high performance in detecting and localizing cyber-attacks.

## 1. Introduction

Smart water distribution systems (WDSs) produce a large amount of information during their operation. This information contains sensor readings, actuator status, and transitions between controllers and programmable logic controllers (PLCs). In WDSs, these data are gathered and synchronized under the supervisory control and data acquisition (SCADA) system. SCADA systems facilitate advanced automation for increasing the system’s efficiency via real-time management and, thus, achieve cost and labor savings while meeting consumers’ demands with higher reliability. However, with the advantages of smart WDSs, a new vulnerability to cyber-attacks has emerged. Due to the automated nature of these systems, communication can be compromised, and operational information and mechanisms might leak. A critical review done by [1] reveals cyber-attack incidents in the water sector in different regions around the globe, including the United States, Europe, the United Kingdom, and Australia. In their review, the authors found that attackers infiltrated the system through different components of the smart WDS, such as routers, PLCs, mail servers, and SCADA systems. The attacks involved data manipulation, data theft, computational resource theft, water theft, and environmental pollution. The study in [1] highlights that only reliable and well-documented resources were reviewed, implying that further undocumented attacks might have occurred.

Cyber protection is needed to overcome this potential vulnerability. Traditional information protection (e.g., firewalls) is vital for isolating the operational network from the business network. Still, an additional protection layer can be gained by analyzing the system’s operational data to identify anomalous behavior, which the attacker is expected to leave in the system [2]. That is, the operational data of the smart WDSs can be used for anomaly detection and specifically for cyber-attack detection. While anomaly detection can help operators detect operational anomalies, like equipment failure or pattern changes, detecting cyber-attacks is more challenging since, unlike normal failures, attackers might try to conceal their actions by altering various parameters. Hence, detecting cyber-attacks has been an active research topic in recent years, as detailed below.

Tuptuk et al. [3] report that researchers’ awareness of cyber-attacks on water systems has significantly increased worldwide in recent years. This is also emphasized in a recent study by Shapira et al. [4], which reviewed stakeholders’ perspectives on cyber risks in the water sector. One notable benchmark is the BATADAL competition [5], in which research teams were challenged to develop the best detection algorithm for detecting cyber-attacks on a given simulated WDS. The datasets provided to the teams included sensor readings from normal operations and a second dataset with operation readings under simulated cyber-attacks (simulated using the epanetCPA toolbox [6]). Seven teams participated in the competition and presented different cyber detection systems. Six out of seven teams in the competition [7,8,9,10,11,12] developed detection algorithms based on statistical methods and supervised machine learning (ML) techniques.

Nonetheless, supervised methods require labeled attack records which are rarely available in real-world applications. One team (the winning team) used a model-based approach that simulated the entire physical system using a hydraulic simulator [13]. It then used a supervised ML method to identify outliers. As such, the proposed model-based approach was impractical for two reasons: (1) it is a supervised method, and (2) it requires a detailed hydraulic model of WDSs, which is often unavailable in real-world systems. It is noteworthy that using simulated cyber events is not a remedy for supervised ML approaches since simulating the cyber events will require a calibrated epanetCPA model [6], which, as explained previously, is often unavailable in the real world. In addition, in the case of producing such attacks, these will only be a few scenarios out of infinite possibilities. Thus, for a detection model to be practical, it should be able to learn the abnormality in WDSs without referring to a predetermined list of labeled attacks. None of the aforementioned studies meets this condition.

In general, anomaly detection over a time series is a challenging problem where the detection system is asked to pinpoint anomalous samples from a real-time data stream. Anomaly detection problems are typically characterized by unbalanced data, meaning most of the samples in the data belong to the ‘normal’ state. In contrast, ‘anomalous’ samples are rare, making detecting these anomalies an even more complex problem. ML algorithms were found suitable to deal with such problems [14]. These algorithms, which are usually named classifiers, will first learn a representation of the system’s state(s) from training samples, and then the trained classifier will be used to classify (or predict) the classes of the new samples. In the context of cyber-attack detection in WDSs, most of the records from the water system represent normal hydraulic behavior, while the records at the time of the cyber-attack represent anomalous behavior. The classifier aims to infer abnormal records from the data stream in real-time.

In general, there are three paradigms that differ in the level of required information during the training stage: (a) supervised-learning classifiers require labels from the classes we wish to predict; in our context, the training data should include a flag for normal/under-attack for each observation in the training dataset, and based on these labels the supervised classifier learns the relation between the samples and the labels; (b) unsupervised-learning classifiers are designed to learn the state of the data without the need of labeled samples. These classifiers attempt to predict the class of new samples based on the learned characteristics of the system in the training stage and (c) semi-supervised learning, which takes advantage of both supervised and unsupervised learning methods. In semi-supervised learning, the classifier requires training datasets that are guaranteed to be clean from anomalies; thus, unlike supervised learning, it does not require flags of normal/under-attack for each observation. Instead, in the semi-supervised learning paradigm, new samples are introduced to a classifier, which only learned the characteristics of the clean samples and so can distinguish anomalous samples when they do not fit the learned patterns [15].

Following the BATADAL competition, several papers suggested using deep learning (DL) techniques for the cyber-attack detection problem in smart WDSs. DL is a research field in ML, which generally aims to learn complex problems using stacked artificial neural networks (ANNs) in multiple layers. These multilayer ANNs are referred to as deep neural networks (DNNs). DL techniques can perform under supervised, unsupervised, and semi-supervised learning conditions [16]. Taormina and Galelli, [17], proposed the use of auto-encoder (AE), a DNN architecture that aims to learn the decompressed representation of the data by encoding and then decoding the input data. This method allows us to learn the normal operation of the WDS by minimizing the reconstruction error between the input and output layers. Then, when the new samples are introduced to the AE, the reconstruction error is used to decide if the sample is normal or not. It is expected that anomalous samples will produce higher reconstruction errors since the model has never seen them before; thus, the decoded output of the samples will be very different from the input samples for the anomalous input. The authors conducted a thorough sensitivity analysis using labeled attack datasets to choose the optimal compression factor, hidden layers’ dimensions, and ANN’s depth. The detection method requires two tuning parameters: error threshold and smoothing window size. Utilizing sensitivity analysis, the authors showed that several selections of tuning parameters performed well under the two different labeled datasets provided in the BATADAL competition. Following this observation, the authors recommended tuning parameters. If the values of the recommended tuning parameters are adopted without change, the suggested method could be classified as a semi-supervised method since it relies solely on the clean training dataset.

Nonetheless, the sensitivity analysis was performed on the two labeled datasets that belong to the same WDS network (C-Town). As such, it is unclear whether the recommended tuning parameters generalize to other networks. If not, a new tuning will be required, and the suggested method will be supervised. Additionally, the study in [17] proposed a scheme for event localization, but part of this scheme requires manual intervention of expert analysis.

Another DL algorithm, suggested in [10], proposed using a variational autoencoder (VAE). Unlike the auto-encoder used in [17], which learns the compressed representation of the data, the VAE learns the distribution of the data. The suggested ANN architecture is more complex and deeper. The authors use the log-reconstruction probability function (LPR) to identify anomalies. The authors set their LPR thresholds using an enumeration process to maximize the performance, which puts the method under the supervised learning category.

Kadosh et al. [18] suggested the one class detection system (OCDS), which uses one class of classifier (support vector data description (SVDD)) trained on normal data. The output of the SVDD is classified into abnormal/normal data while utilizing a decision rule which requires two tuning parameters. These parameters are calibrated using labeled datasets. As such, this approach is classified as supervised learning. Hence, as a supervised approach, the OCDS might be impractical for real-world applications which lack labeled-attacks datasets.

In this study, we propose the semi-supervised detection system (SSDS), which is fully automatic and can be generalized to different water networks and datasets without needing labeled attacks. Unlike previous studies, we show one configuration of the classification process, which yields high performance in different networks and datasets. Furthermore, most previous studies have developed detection methods without considering the localization problem [3]. This study addresses this gap by developing a localization methodology that almost guarantees that the attacked network zone is identified.

The distinct feature of the SSDS is that it relies on semi-supervised learning; thus, it does not require labeled attacks in the training dataset. The SSDS relies on the multivariate correlation between different sensor types using maximum canonical correlation analysis (MCCA) to achieve a dimensional reduction of the problem. This reduced representation is then classified using a semi-supervised method such that the entire learning process depends solely on the attack-free training dataset. The methodology depends on three modules that test different aspects of the WDS governing physics. The first module analyzes the physical relationships within a specific zone in the WDS (e.g., DMA), the second analyzes the temporal trends of the data, and the third examines the relationships between the different zones. The insights from the three modules are synthesized to detect and localize cyber-attacks.

To demonstrate the generalized applicability of the proposed solution, it was tested on two different WDSs and different datasets. In the following sections, we will discuss each building block of the SSDS, our results on the different datasets, and the localization abilities of the SSDS. Finally, in Appendix A, Appendix B and Appendix C, we provide a thorough sensitivity analysis that demonstrates the individual contribution of each module, the impact of parameter change, and data exposure percentage on the overall performance of the SSDS.

## 2. Methodology

### 2.1. Problem Statement

Real-time SCADA records in modern WDSs can be used to develop anomaly-detection algorithms, where historical SCADA data can be used for training the detection model. Often, these historical data contain no records of labeled cyber-attacks; thus, it is impractical to assume the availability of labeled cyber-attack datasets for the training phase. Furthermore, it is impractical to assume that datasets with labeled cyber-attacks could be synthetically simulated because such a process will require a calibrated hydraulic model, which is rarely available in real-life systems. In light of the above, there is a need to develop a practical detection model that only relies on attack-free datasets in the training phase.

### 2.2. Data Preparation

In the semi-supervised learning paradigm, the collected attack-free historical readings are used to train the classification model, and the trained model is then used to classify future readings into normal and under-attack states. Here we considered that the sensors could measure water levels in the storage tanks, water flows in the pumps and valves, and pressure in chosen junctions (e.g., before and after the pumping station). Let $S^{tr}\in {ℜ}^{n\times n_{s}}$ represents attack-free historical readings that will be used for training the model, where n is the number of historical records and $n_{s}$ is the number of sensors. Let $S_{1:t}^{ts}$ be a matrix that contains new SCADA readings after the training stage, where $S_{1:t}^{ts}\in {ℜ}^{t\times n_{s}}$ and 1:t are time (dynamic) indices representing timesteps from the beginning of the system implementation to the current timestep t. New samples are added to $S_{1:t}^{ts}$ at each timestep, and thus rows’ dimension of $S_{1:t}^{ts}$ grows over time. It is noteworthy that the samples in $S_{1:t}^{ts}$ are considered as testing samples, which are not used to train classifiers or tune any parameter in the methodology.

Modern WDSs are usually divided into district metering areas (DMAs) that allow for efficient water loss management and monitoring [19]. Moreover, DMA design allows for efficient management of failure situations, for example, by isolating certain regions and reconfiguring water routes in case of low pressure due to pipe bursts. Kadosh et al. [18] showed that the DMA structure could be utilized to design ML anomaly detection, which accounts for some of the physical properties of WDSs. More specifically, assume we have a set of DMAs, where d∈DMAs, then we can define $S^{tr,d}$ and $S_{1:t}^{ts,d}$, which contain train and test data from a specific DMA.

### 2.3. Support Vector Data Description

**Figure 1:**
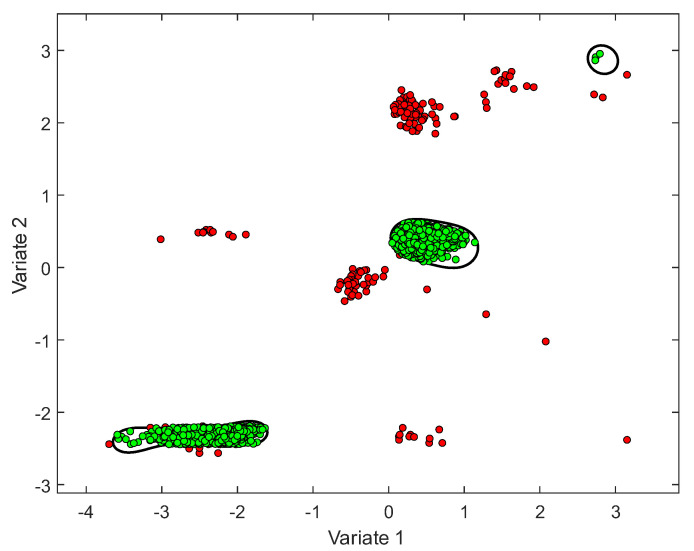
Illustration of two variates classified by SVDD boundaries (in black curves). Green points are inliers and red points are outliers.

Support vector data description (SVDD) is a one-class classifier algorithm suggested by Tax and Duin [20]. The SVDD fits a boundary around one predefined class (e.g., normal observations class). When new data are introduced, this boundary can be used later for classification purposes. That is, samples that fall outside the boundary can be considered outliers by the SVDD classifier (Figure 1). To construct the boundary, the SVDD uses a radial-basis-function (RBF) kernel, which is parametrized by the tuning parameter γ. Another parameter that impacts the shape of the boundary is the C parameter, which is known as the penalty constant, where $\frac{1}{n}\leq C\leq 1$, and n is the number of samples in the training set [21]. The C parameter controls the trade-off between the volume inside the boundary and the excepted outliers. Setting C=1 means that we assume all samples belong to the normal class and there are no anomalous samples present in the training data. Thus, setting C=1 agrees with our previous assumption regarding the attack-free training dataset. Under this setting, the SVDD solves the following optimization problem to construct the boundary [21]:

(1) $$\begin{array}{l} \underset{\;\;\;\;\;\;\;\;\;\;\;\;\;\;\;\;\;\;\;\;R,a}{\;\;\;\;\;\;\;\;\min}\;\;\;R\;\;\;\;\\ s.t.\;\;\;\;\left\|\phi (x_{i},\gamma )-a\right\|\leq R \end{array}$$

where R is the radius of the boundary, a is the center’s coordinates, and $\phi (x_{i},\gamma )$ is the mapping function (e.g., RBF). When no outliers are expected, the inequality constraint is treated as an equality constraint, and the Lagrange multipliers method can be used to solve the problem.

After constructing a data boundary by solving the optimization problem in Equation (1), a decision value (DV) could be obtained for any sample. The sample is on the boundary when DV=0, inside the boundary when DV<0, and outside the boundary when DV>0.

In light of the above, we are left with the parameter γ as the only tuning parameter for the SVDD. The γ parameter controls the bandwidth of the SVDD boundary when using the RBF kernel. As opposed to C, the tuning process for this parameter is not trivial. Setting it to a large value can lead to overfitting, and setting it to a small value can lead to underfitting (i.e., the boundary will be far away from the data cloud). The studies in [22,23,24] explored unsupervised tuning methods for γ selection when using SVDD with RBF kernel. We adopted the modified mean criterion method as suggested by [24] since it can be computed fast and has been validated on different datasets [24]. The modified mean criterion is given in Equation (2):

(2) $$\gamma =\frac{\left(n-1)\ln(\frac{n-1}{\delta^{2}}\right)}{2n\sum_{i=1}^{m}\sigma_{i}^{2}}$$

where n is the number of samples, m is the number of learned features, $\sigma_{i}^{2}$ is the variance of the $i^{\mathrm{th}}$ feature, and δ is a tolerance factor. As suggested by [24], the value of the tolerance factor δ is the root of the function f(δ), as given in Equation (3):

(3) $$f(\delta )={\left[\ln(n-1)-2\ln(\delta )\right]}^{-\frac{3}{2}}-\delta$$

### 2.4. Ensemble Classification

Assigning only one SVDD classifier to learn the entire SCADA readings can lead to misclassification. We found that as the dimensionality of the data grows and the number of samples gets higher, the performance of the SVDD drops. To overcome this issue, we used ensemble learning [25,26]. The idea of ensemble methods in ML is to combine more than one classifier to handle the same problem. Later, in the prediction phase, predictions from individual classifiers are combined using a fusion operator. We rely on the previous observations of Kadosh et al. [18], in which they suggest using the physical understanding of the water network to train different classifiers. Kadosh et al. [18] suggested that assigning an SVDD classifier to each DMA separately can dramatically improve the results; thus, a specific classifier is trained for each DMA. In this paper, we extend this idea by assigning dedicated classifiers that examine the physical relationships between flow, storage, and pressure, which are known to exist in WDSs. Specifically, for each DMA, we perform within-DMA spatial analysis (WSA), which trains different classifiers for detecting anomalies between different combinations of physical variables. For example, we train a specific classifier for the interaction between flow-pressure variables, a different one for the interaction between flow-storage variables, etc. In addition to the WSA, we perform between-DMAs spatial analysis (BSA). This analysis trains dedicated classifiers that check anomalies between pairs of DMAs. As such, the analysis will be capable of detecting anomalies when one of the DMAs behaves abnormally compared to other DMAs.

Moreover, it is expected that WDSs will have normal temporal trends that can be used to detect anomalies when the operational data deviate from previously observed temporal patterns. To capture this deviation, we perform within-DMA temporal analysis (WTA), in which dedicated classifiers are trained to detect anomalies between DMA features and temporal regime curves that characterize the expected temporal pattern in the DMA. As such, an Ensemble of classifications will be obtained from the three modules (WSA, BSA, and WTA). These classifications are used to predict the system’s final state as being under attack or not. To perform the attack detection and localization synthesis, we rely on the fusion operators described in Section 2.9. Following the framework above, one needs to train a large number of different classifiers in the three modules while focusing on the physical relationships between flow, pressure, and storage variables. To capture these physical relationships, we propose maximum canonical correlation analysis (MCCA).

### 2.5. Maximum Canonical Correlation Analysis

The WDS’s water mass and energy balance govern the relationship between flow, pressure, and storage variables. This relationship is complex (nonlinear and discontinuous) and depends on the hydraulic properties of the network (pipe resistance, pump curves, etc.). A good approximation can be obtained by hydraulic simulators such as EPANET [27], but this requires a calibrated simulator, which is often unavailable in real-life systems [13]. Previous studies suggested using deep learning methods to characterize the hydraulic variables’ nonlinearity and complexity [10,17,28]. Here, we suggest a more straightforward approach that relies on dimensionality reduction and linear approximation using MCCA coupled with SVDD classifiers. Specifically, we seek the best linear association between the two groups of variables (for example, flow vs. pressure) by linearly projecting the features from high dimension to 2D. Classifiers will then be trained to classify the 2D projections of the features.

MCCA could be considered as the multivariate extension of correlation analysis. For example, when we have two scalar random variables, each with n observations (i.e., $x\in {ℜ}^{n\times 1}$, $y\in {ℜ}^{n\times 1}$), then Pearson correlation could be calculated as ρ(x,y) to measure the degree of linear association between them. However, when having two vectors of random variables, each with n observations (i.e., $X\in {ℜ}^{n\times m_{1}}$, $Y\in {ℜ}^{n\times m_{2}}$), then MCCA seeks linear combinations of X and Y which have a maximum correlation with each other. That is, we solve the optimization problem in Equation (4):

(4) $$\max_{a,b}\rho (X\cdot a,Y\cdot b)$$

where $a\in {ℜ}^{m_{1}\times 1}$ and $b\in {ℜ}^{m_{2}\times 1}$ are vectors of coefficients to project the correlation problem from dimensions $m_{1}$ and $m_{2}$ to a classical 2D correlation problem. The above problem could be solved using singular value decomposition on a correlation matrix [29] and is implemented in many programming languages such as MATLAB and R.

Upon finding the optimal a and b, we can linearly project the high dimensional data into the 2D domain [30]. In canonical correlation terminology, we distinguish between the terms ‘variables’ and ‘variates’. The term ‘variables’ refers to the original variables, while the term ‘variates’ refers to the projected variables.

We used the above MCCA procedure to find the best linear association between groups of physical variables based on governing physical rules. The definition of X and Y in our context will change based on the physical relationship we are trying to capture. For example, if we want to capture the energy-balance relationship between flow and pressure inside a given DMA, then X will be defined as the samples from the flow sensors and Y will be defined as the samples from the pressure sensors of the DMA. On the other hand, if we want to capture the water-balance relationship between flow and storage inside a given DMA, then X will be defined as the samples from the flow sensors and Y will be defined as the samples from the tank storage sensors of the DMA. We also consider other combinations, as detailed in the following sections. It is expected that the linear approximation will not yield high accuracy when high nonlinearity exists. Yet, our methodology does not require the linear approximation to be very accurate since the SVDD will be able to characterize the expected error level in the linear approximation, as we will demonstrate in the application.

In the following sections, we present the offline stage in which the variates of the MCCA are derived and used to train the SVDD classifiers based on historical data. The offline stage is followed by an online stage where the variates are used in the classifiers to predict the system state. Finally, in Section 2.9, we show how we can synthesize the different classifiers’ predictions to identify the anomalous behavior of the system.

### 2.6. Module 1: Within-DMA Spatial Analysis (WSA)

**Figure 2:**
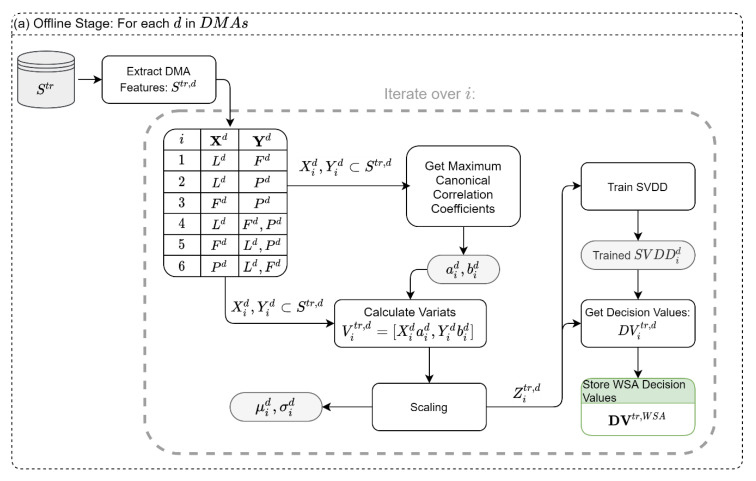

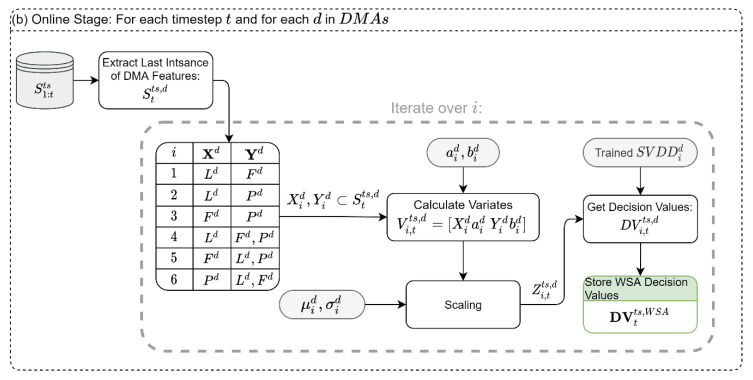
Within-DMA spatial analysis module: (**a**) Offline Stage, (**b**) Online stage. Notes: Gray shade is the data created in the Offline Stage and used in the Online Stage; green shade is the data used in the synthesis module.

The WSA module aims to find anomalies between three sensor types inside a DMA d: (1) water level (storage) sensors $L^{d}$, (2) flow sensors $F^{d}$, and (3) pressure sensors $P^{d}$. The WSA module operates in offline and online modes. In the offline mode, we rely on the attack-free dataset $S^{tr,d}$ to find the variates that yield the maximum correlations between sensor types, and then based on these variates, a 2D-SVDD classifier is trained (Figure 2a). The steps of the offline module are as follows: first, the historical sensor readings of a specific DMA, $S^{tr,d}$, are loaded from the training set $S^{tr}$; then, the sensors are divided into six possible type-combinations (interactions between sensors type), $X_{i},Y_{i}\;\;\;\forall i=1...6$. The six combinations are the result of one sensor type against a different sensor type (three combinations, $\left\{L^{d}\}-\{F^{d}\right\}$, $\left\{L^{d}\}-\{P^{d}\right\}$, $\left\{F^{d}\}-\{P^{d}\right\}$) and one sensor type against the rest of the types (three combinations, $\left\{L^{d}\}-\{F^{d},P^{d}\right\}$, $\left\{F^{d}\}-\{L^{d},P^{d}\right\}$, $\left\{P^{d}\}-\{L^{d},F^{d}\right\}$). Then, MCCA is used to extract the maximum correlation between each set, and the coefficient vectors, $a_{i}^{d},b_{i}^{d}$, are stored for later use. The variates of the MCCA are then scaled to a zero mean and unit standard deviation and used to train a 2D-SVDD model for each combination, $SVDD_{i}^{d}$. The trained SVDDs, the mean, and the standard deviation values of the scaling process are stored for later use in the online stage.

Moreover, the scaled variates, $Z_{i}^{tr,d}$, are used to derive the “normal” decision values (DVs) that are expected from each SVDD. The vast majority of the DVs will be negative values (i.e., normal state) since we are predicting the same attack-free data used to construct the SVDD boundary. However, the DVs might include small positive values resulting from a small violation of the SVDD boundary in some samples (for example, because of a precision threshold). As such, it is important to calculate the DVs in the training stage to assess the magnitude of the positive DVs under attack-free conditions. The entire process is looped through all six types of combinations and all DMAs, as shown in Figure 2a.

To demonstrate the above, let us consider a DMA (d=1) which has 100 observations from 3 flow sensors, 4 pressure sensors, and 2 water level sensors. In the first combination, $i=1:\;\;\;\{L^{d=1}\}-\{F^{d=1}\}$, we use the MCCA to find vectors of the coefficients $a_{1}^{1}\in {ℜ}^{2\times 1}$ and $b_{1}^{1}\in {ℜ}^{3\times 1}$ that maximize the linear association $\;\rho (X_{1}^{1}\cdot a_{1}^{1},Y_{1}^{1}\cdot b_{1}^{1})$, where $X_{1}^{1}\in {ℜ}^{100\times 2}$ contains the water level data, and $Y_{1}^{1}\in {ℜ}^{100\times 3}$ contains the flow data. In the sixth combination, $i=6:\;\;\;\{P^{d=1}\}-\{L^{d=1},F^{d=1}\}$, we use the MCCA to find vectors of the coefficients $a_{6}^{1}\in {ℜ}^{4\times 1}$ and $b_{6}^{1}\in {ℜ}^{5\times 1}$ that maximize the linear association $\;\rho (X_{6}^{1}\cdot a_{6}^{1},Y_{6}^{1}\cdot b_{6}^{1})$, where $X_{6}^{1}\in {ℜ}^{100\times 4}$ contains the pressure data, and $Y_{6}^{1}\in {ℜ}^{100\times 5}$ contains both the water level and flow data. Similarly, we can define the rest of the type combinations (i=2...5). Note that regardless of the dimensions of X and Y, we will always obtain two variates after projecting the samples using the obtained coefficients from the MCCA. These two variates are used to train a 2D SVDD. Figure 1 demonstrates the SVDD classification in 2D after projecting the high-dimensional samples into two variates.

It is noteworthy that the WSA (and the BSA that will be discussed later) focuses on the spatial analyses of the data in a single timestep. As such, there is an inherent assumption of time series stationarity for all sensors. If one of the sensors has a nonstationary time series, this can be converted into a stationary one by differentiating it (i.e., the difference of observations in successive timesteps). To check whether differentiation should be performed on the sensor data, the Kolmogorov–Smirnov test [31] is used to pinpoint situations when the probability distributions of the first and the second half of the training dataset differ.

In the online stage of the WSA module, the parameters and the SVDD classifiers obtained in the offline stage are applied to the test dataset, $S_{1:t}^{ts,d}$, as illustrated in Figure 2b. In this stage, samples are fed to the system in real-time; this is demonstrated in Figure 2b by the growing index t in the dataset, from which we only rely on the last instance $S_{t}^{ts,d}$. Then, using the scaling parameters and the vectors of the MCCA coefficients from the offline stage, the scaled variates, $Z_{i,t}^{ts,d}$, are obtained. These variates are then classified using the trained SVDD models, $SVDD_{i}^{d}$, and the DVs are stored for the synthesis module. It is noteworthy that for each time instance t, each DMA will result in six DVs corresponding to the six type combinations.

### 2.7. Module 2: Within-DMA Temporal Analysis (WTA)

**Figure 3:**
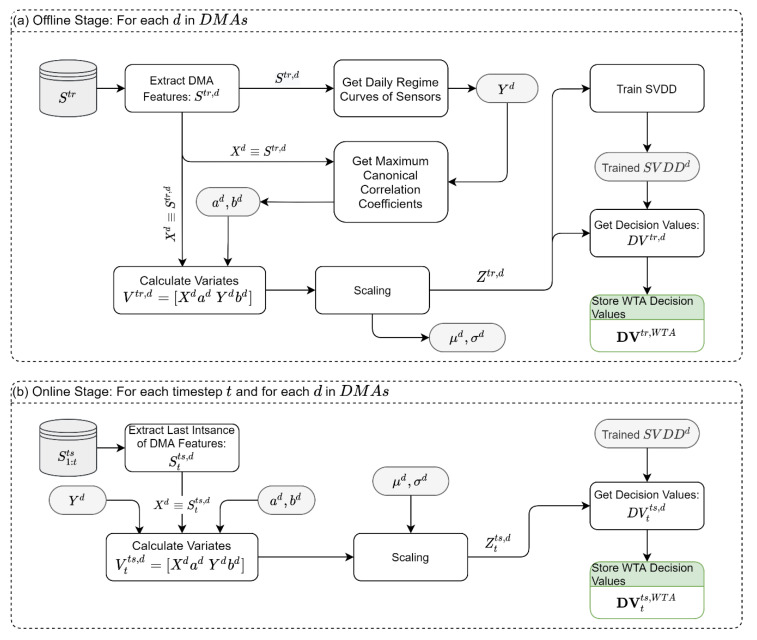
Within-DMA temporal analysis module: (**a**) Offline Stage, (**b**) Online Stage. Notes: Gray shade is the data created in the Offline Stage and used in the Online Stage; green shade is the data used in the synthesis module.

The WTA module aims to detect anomalies in the temporal patterns observed in the historical data (Figure 3). To capture the time pattern, we use daily regime curves of the sensory data. The daily regime curves are calculated by averaging the sensor data for each hour of the day. That is, each hour in the day (1 to 24) will take a unique representative (reference) value.

In the offline stage of the WTA (Figure 3a), we compare the sensor readings (of all types) to their respective daily regime curve using the MCCA. Under this setting, the MCCA finds the vectors of coefficients $a_{}^{d},b_{}^{d}$, that yield the maximum correlation $\;\rho (X_{}^{d}\cdot a_{}^{d},Y_{}^{d}\cdot b_{}^{d})$, where $X^{d}$ is all sensor readings from DMA d and $Y^{d}$ is the corresponding daily regime curves of the sensors. The variates of the MCCA are then scaled to a zero mean and unit standard deviation and used to train a 2D-SVDD model for each DMA, $SVDD_{}^{d}$. The trained SVDDs, the MCCA coefficients, the regime curves, the mean, and the standard deviation values are stored for later use in the online stage. Moreover, the scaled variates, $Z_{}^{tr,d}$, are used to derive the “normal” DVs that are expected from each SVDD. In the online stage (Figure 3b), the samples are fed to the system in real-time, and we only rely on the last observations $S_{t}^{ts,d}$. Using the scaling parameters, the regime curves, and the vectors of MCCA coefficients from the offline stage, the scaled variates, $Z_{t}^{ts,d}$, are obtained. These variates are then classified using the trained SVDD models, $SVDD_{}^{d}$, and the DVs are stored for the synthesis module.

To demonstrate the above, let us consider a DMA (d=1) which has 100 observations from 3 flow sensors, 4 pressure sensors, and 2 storage sensors. We use the MCCA to find the vectors of coefficients $a_{}^{1}\in {ℜ}^{9\times 1}$ and $b_{}^{1}\in {ℜ}^{9\times 1}$ that maximize the linear association $\;\rho (X_{}^{1}\cdot a_{}^{1},Y_{}^{1}\cdot b_{}^{1})$, where $X_{}^{1}\in {ℜ}^{100\times 9}$ contains all sensory data and $Y_{}^{1}\in {ℜ}^{100\times 9}$ contains the daily regime curves of all sensory data. Regardless of the dimensions of X and Y, we will always obtain two variates after projecting the samples using the coefficients from MCCA. These two variates are used to train a 2D SVDD in the offline stage and to predict the classification in the online stage.

### 2.8. Module 3: Between-DMA Spatial Analysis (BSA)

**Figure 4:**
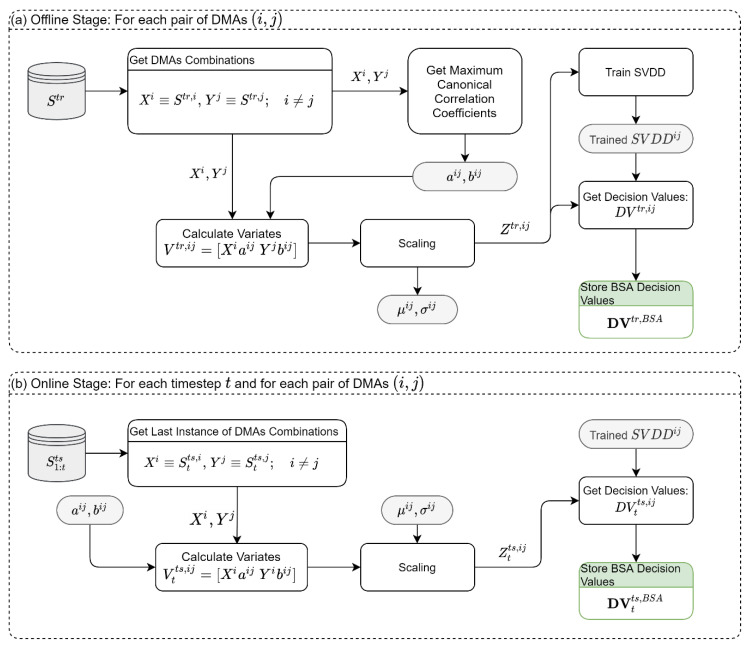
Between-DMA spatial analysis module: (**a**) Offline Stage, (**b**) Online Stage. Notes: Gray shade is the data created in the Offline Stage and used in the Online Stage; Green shade is the data used in the synthesis module.

Unlike the WSA, which focuses on anomalies within DMAs, the BSA focuses on anomalies between pairs of DMAs (i,j) as shown in Figure 4. The BSA module builds on the idea that modern WDSs are usually partitioned into DMAs, and that there is an intrinsic correlation between DMAs due to sharing different hydraulic components. For example, multiple DMAs can share a water tank, or a large DMA can feed nearby DMAs. In this module, we use the MCCA to extract the maximum correlation between the sensory readings (all types) of DMA i, $X^{i}$, and the sensory readings of DMA j, $Y^{j}$. Then we follow similar steps as in the previous modules. But unlike the previous modules, in the BSA, the analysis is performed for each pair of DMAs. For example, in a network with five DMAs, we need to apply the MCCA 5×4/2=10 times to extract 10 vectors of the coefficients $a^{ij},b^{ij}$, and to train 10 SVDDs on the variates.

### 2.9. Synthesis

**Figure 5:**
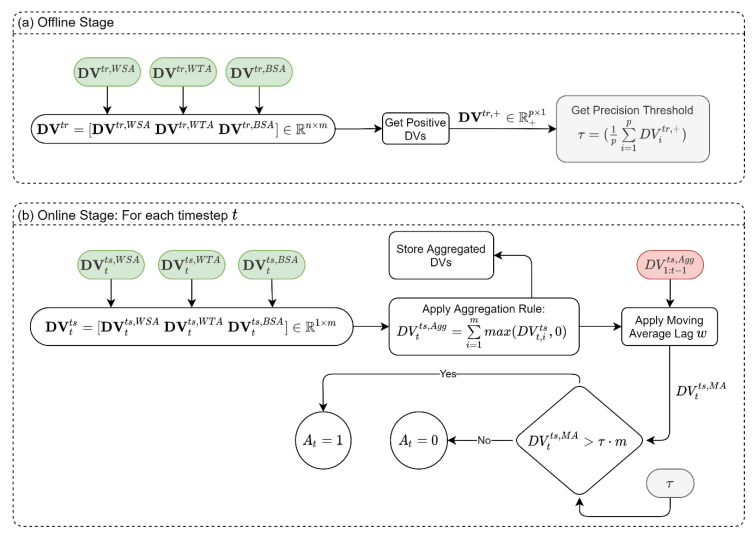
SSDS synthesis and detection process: (**a**) Offline Stage, (**b**) Online Stage. Notes: Gray shade is the data created in the offline stage and used in the online stage; Red shade is the data saved in previous timesteps; Green shade is the data used in the synthesis module from previous modules.

The previously described modules result in multiple SVDDs, each reporting its own DV, where positive DVs indicate outliers for the performed analysis. In the synthesis stage, we want to reach the final identification of the detection system state (Alarm, $A_{t}=1$ versus not Alarm, $A_{t}=0$) based on all the DVs obtained from the classifiers ensemble. That is, we need to develop an aggregation rule for all DVs. Figure 5 shows the synthesis module, which is also divided into offline and online stages. In the offline stage, we estimate the average precision of the SVDDs. As explained previously, the SVDDs are expected to produce small positive DVs even when tested for “normal” data. This can be attributed to the precision threshold in the SVDD training process. Therefore, we can estimate the mean precision by using the DVs that were stored in the training stage of the three modules (see green boxes in Figure 2a, Figure 3a and Figure 4a). To do so, the DVs from the three modules $\left[DV_{}^{tr,WSA},DV_{}^{tr,WTA},DV_{}^{tr,BSA}\right]$ are joined together to form a matrix of DVs, $DV^{tr}\in {ℜ}^{n\times m}$, where n is the number of observations in the training dataset and m is the number of all SVDDs in the three modules. To estimate the average precision, τ, we average the positive entries in $DV^{tr}$, as shown in Figure 5a. In the online stage, samples are fed to the system in real-time, while we only rely on the last DVs obtained from the three modules, which is a vector, $DV_{t}^{ts}\in {ℜ}^{1\times m}$, that aggregates the last updated DVs of the three modules $\left[DV_{t}^{ts,WSA},DV_{t}^{ts,WTA},DV_{t}^{ts,BSA}\right]$. We then use the aggregation rule in Equation (5) to get a single scalar DV for timestep t.

(5) $$DV_{t}^{ts,Agg}=\sum_{i=1}^{m}\max(DV_{t,i}^{ts},0)$$

The replacement of negative values with zero is performed to outweigh inlier samples in the aggregation operation. We also store all the aggregated DVs up to time t in a vector denoted $DV_{1:t-1}^{ts,Agg}$. In the next step, we perform a moving average with lag w on the aggregated DV to obtain the moving average value $DV_{t}^{ts,MA}$. Finally, to declare an alarm, $DV_{t}^{ts,MA}$ is compared to the precision of the SVDDs. If it exceeds the precision, then an alarm is raised. Note that $DV_{t}^{ts,MA}$ is compared to average precision τ multiplied by m; this is because the DV values in $DV_{t}^{ts,MA}$ result from a summation of m terms, as can be seen in Equation (5).

### 2.10. Performance Measures

Given a vector of alarm indicators, A (a vector that collects $A_{t}\;\;\forall t$), from a testing dataset, we can examine the system’s performance against labeled attacks. It is noteworthy that, unlike supervised learning methodologies, labeled attacks are only used for testing purposes. First, we consider the score index, S, as defined in BATADAL [5]:

(6) $$S=\frac{S_{TTD}+S_{CM}}{2}$$

where $S_{TTD}$ is time-to-detection score, defined as:

(7) $$S_{TTD}=1-\frac{1}{n_{a}}\sum_{i}^{n_{a}}\frac{TTD_{i}}{\Delta t_{i}}$$

where $n_{a}$ is the number of attacks contained in the dataset, $TTD_{i}$ is time-to-detection relative to the *i*^th^ attack, and $\Delta t_{i}$ is the duration of the *i*^th^ attack. $S_{CM}$ is the classification score:

(8) $$S_{CM}=\frac{TPR+TNR}{2}$$

where TPR=TP/(TP+FN) is the true positive rate or the recall and TNR=TN/(TN+FP) is the true negative rate. True positive (TP) is the number of under-attack instances detected by the algorithm, true negative (TN) is the number of non-event instances (i.e., safe state), which are also reported as safe by the algorithm, false negative (FN) is the number of under attack instances missed by the algorithm, and false positive (FP) is the number of instances which the algorithm flagged as under attack, but were actually safe. $S_{CM}$ varies between 0 and 1, with $S_{CM}=1$ being the ideal case in which all attacks are detected with no false alarms. The study in [5] argued that the S score might be biased and suggested the $F_{1}$ score. As such, in addition to S score, we measured the performance with $F_{1}$ score, which is suitable for imbalanced data [32]. $F_{1}$ is defined as follows:

(9) $$F_{1}=2\frac{PPV\cdot TPR}{PPV+TPR}=\frac{TP}{TP+0.5\cdot (FP+FN)}$$

where PPV is the positive predicted value or precision and defined as *TP/(TP + FP)*. The advantage of $F_{1}$ when scoring the detection of rare events is that it does not depend on the number of TN samples, which is expected to be high in unbalanced data with a large number of normal samples. For comparison with previously published studies, we report both S and $F_{1}$.

## 3. Results

The SSDS was developed using MATLAB R2020a (the code and data are provided on GitHub, https://github.com/SWSLAB/SSDS, accessed on 4 August 2022). For implementing the SVDD classifiers, we compiled the C++ source code of LIBSVM [33] on a 64-bit Windows 10 OS. The SSDS is tested with w=12 in all runs.

To compare the performance of the suggested algorithm with previous algorithms in the literature and to demonstrate the generalization of the SSDS to different networks, we tested it on two different case studies, C-Town [5] and E-Town [18]. The two case studies are simulated SCADA readings of WDSs, containing a clean training dataset and two datasets with labeled cyber-attacks. Both case studies were simulated using the epanetCPA toolbox [6], which can simulate cyber-attacks on the cyber layer of the system, such as sensors, actuators, PLCs, and SCADA systems. Unlike many previous studies that used one of the datasets with labeled attacks to calibrate the detection system using a supervised learning method, here we only use the two labeled datasets for testing purposes.

### 3.1. C-Town Case Study

**Figure 6:**
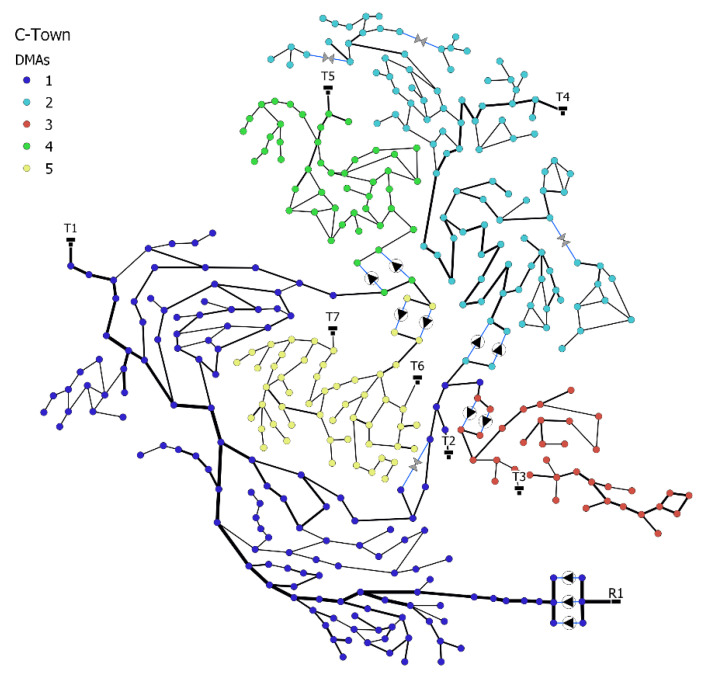
C-town WDS layout, DMAs by colored nodes, R1 is the water source, T1–T7 are the storage tanks, black triangles are pumps, gray triangles are valves, and pipe diameter is illustrated by the line thickness.

The C-Town case study (Figure 6) was first introduced in the BATADAL competition [5]. The case study considers a mid-size WDS, with three datasets capturing 43 sensors in the system, reporting hourly. The datasets include an attack-free training dataset with 8761 samples and two datasets with labeled attacks, denoted as Test 1 and Test 2, each containing seven cyber-attacks. Test 1 has 4177 samples, and Test 2 has 2089 samples. The WDS is divided into five DMAs. Table 1 shows the DMAs and the number of sensors of different types (flow, pressure, and water level) that we consider in the SSDS. It is noteworthy that we excluded sensors that reported constant values in the training dataset.

**Figure 7:**
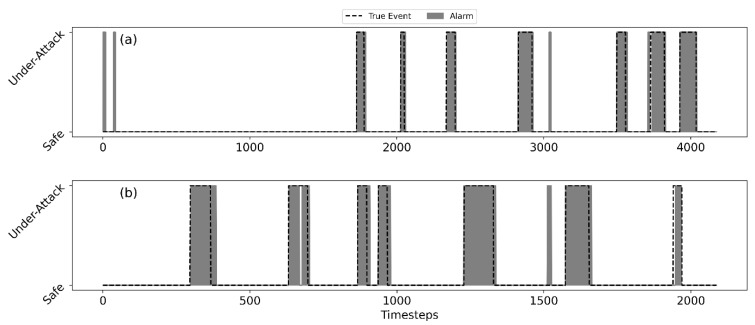
SSDS detection performance; alarms in gray, true events in dashed line; C-Town, Test 1 (**a**), Test 2 (**b**).

Figure 7 shows that the SSDS can capture all attacks in both tests. In Figure 7, the dashed line is the true event, while the gray area indicates the raised alarms. As such, the white area represents true negative samples. The gray area under the dashed line represents true positive detection, while the gray area without the dashed line represents false positive detection. A dashed line with a white area represents false negative samples. The and $F_{1}$ performance measures of the SSDS on BATADAL’s Test 1 and Test 2 are shown in Table 2. A closer look at the raised alarms from Tests 1 and 2 reveals that in most of the events, the alarm continues a few timesteps after the end of the event. An explanation for the false alarms at the end of the events can be attributed to the effect of the smoothing (moving average) function in the synthesis module. Since the smoothing function uses a retrospective moving average, the decision at time instances near the end of the event is still affected by the anomalous DVs obtained during the event. Still, the performance in terms of S and $F_{1}$ scores is high as can be seen in Table 2.

Table 3 compares the results of the SSDS with other previous studies. We found two studies [10,17] that were designed in a semi-supervised fashion. The SSDS outperformed the VAE of [10]; however, when compared to the best result reported by [17] for the AE algorithm, the SSDS shows a lower Precision with a better Recall, ending with a delta of 0.002 below the AE algorithm for the  score. With that said, it is important to mention that we compared our results to the best result reported by [17], in which they conducted a sensitivity analysis of all possible tuning parameters. Therefore, in a way, this result could be interpreted as a result of a supervised algorithm as opposed to the semi-supervised method presented herein. This tips the scale toward our SSDS since the results reported in Table 3 were obtained without any parameter tuning (as shown in the sensitivity analysis). Furthermore, the SSDS shows good performance in the two different networks (as we will see next), while the AE algorithm of [17] was only tested on the C-town network.

Table 3 also compares the SSDS performance with the supervised methods of [7,18]. The SSDS produces very close results to the supervised methods, even though no labeled attacks are used. These results demonstrate that the SSDS is a practical methodology that can compete with other supervised methods that unrealistically assume the availability of labeled attacks.

### 3.2. Attacks Localization

The previous section shows that the SSDS can effectively alert users to an ongoing attack. In this section, we will demonstrate the ability of the SSDS to localize the attack by a voting majority rule. The voting system, which is based on the three modules of the SSDS, can identify the DMA being subjected to a cyber-attack.

**Figure 8:**
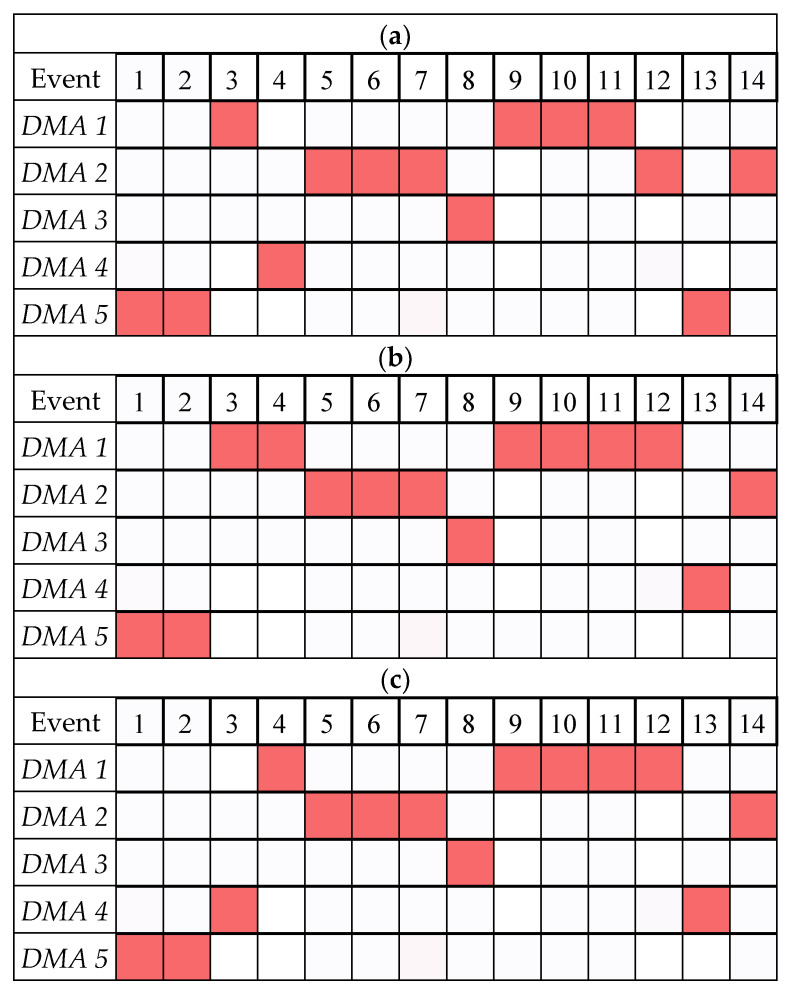
Localization votes by the SSDS modules, illustrating the voting on C-town’s events. (**a**) Localization Votes of the WSA Module. (**b**) Localization Votes of the WTA Module. (**c**) Localization Votes of the BSA Module.

For each detected attack in Figure 8, each of the three modules will sum the positive DVs related to each DMA. Then the DMA with the maximum sum of positive DVs will be declared as the attacked DMA by the module. For example, in the WSA, we sum the positive DVs in $DV_{WSA}^{ts,d}\;\forall d\in DMAs$ during the detected event, and then the WSA module identifies the attacked DMA as the one with the maximum sum of positive DVs. Figure 8a shows the attacked DMAs identified by the WSA module in each of the 14 events in Test 1 and Test 2. Similarly, Figure 8b,c show the attacked DMA identified by the WTA and the BSA modules, respectively.

**Figure 9:**
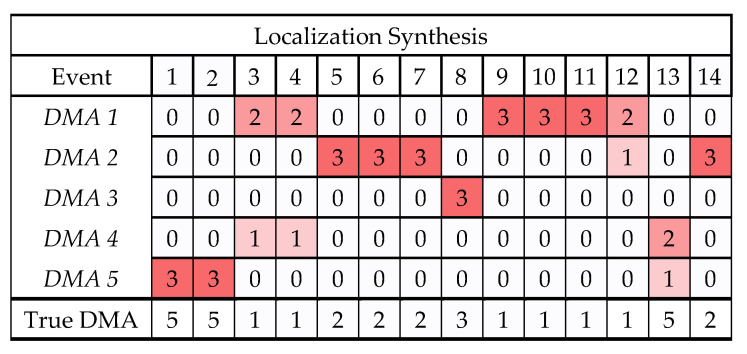
Events localization by aggregated votes from WSA, WTA, and BSA modules in the C-town case study.

The votes from the three modules are aggregated, as shown in Figure 9. Figure 9 shows the votes (out of three) for each DMA and each event. The total number of votes in each event is three, and the DMA that gets the most votes is the most suspected to be the targeted DMA. The last row in Figure 9 also shows the true attacked DMA as detailed by the BATADAL events description [5]. Figure 9 shows that in 10 out of the 14 events, there is a complete consensus between the three modules on the true attacked DMAs. In events 3, 4, and 12, 2 out of 3 modules identified the correct attacked DMA. If we consider the majority voting rule, the only missed attack was event 13, identified as belonging to DMA 4 by two modules and DMA 5 only by the WSA module, while the true DMA was DMA 5.

### 3.3. Sensitivity Analysis

To examine the different components of the suggested SSDS, we conducted three sensitivity analyses, as detailed in Appendix A, Appendix B and Appendix C. In Appendix A, we test the performance of each module of the SSDS. We observed different contributions from the three modules over the different datasets (from the same WDS) and the different networks (C-Town vs. E-Town). This implies that combining the three modules is essential to achieve robust performance. In Appendix B, we assessed the sensitivity of the method to the window size parameter. The results show that w=12 is not ideal for any of the datasets. This implied that tuning the value of w will lead to even higher scores. Nonetheless, the performance was relatively high for a wide range of values. In this study, we used half a day (w=12) without tuning on labeled attacks; this makes our approach semi-supervised. Finally, in Appendix C, we test the performance of the SSDS for different data exposure levels. The results show that the SSDS algorithm can perform well using only 50% of the training datasets.

### 3.4. E-Town Case Study

**Figure 10:**
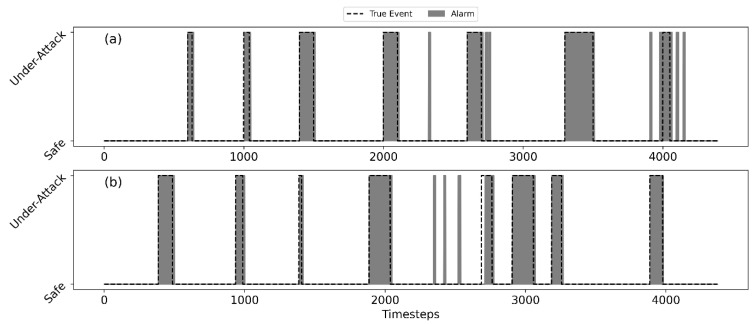
SSDS Detection performance; flags in gray, true events in dashed line. E-Town: Test 1 (**a**) and Test 2 (**b**).

The E-Town case study was first introduced by [18]. The E-Town WDS simulates a realistic water system in a city in Colombia [19]. Compared to C-town, E-Town is a large-scale network, roughly 30 times bigger, including a total of 135 sensors. E-Town includes a training attack-free dataset with 8761 samples and two datasets with labeled attacks, which we denote Test 1 and Test 2. Each testing dataset includes ten attack events, some of which are overlapped and partly overlapped (see [18] for a more detailed description of the attacks). Testing the performance of the SSDS on a large-scale WDS is important to show the scalability of the algorithms while maintaining high performance. The E-Town dataset is also challenging because the samples are simulated over 17,522 h, making a total of two years of readings, during which the system’s normal behavior changes. Namely, attack-free sensor readings can change their distribution over time; thus, the detection algorithm must be robust enough to deal with the distributional change of normal readings. E-Town is divided into 23 DMAs as detailed by [18]. The SSDS disclosed all events in Test 1 and Test 2, as shown in Figure 10. The performance scores of the SSDS on the E-Town case study are detailed in Table 2. Examining the alarms in E-Town’s tests shows a slight delay in the alarm at the beginning of the events, while at the end of the events, the alarm lasts a few timesteps after the events end. As explained previously, this could be attributed to the retrospective moving average in the synthesis module. Yet, the performance scores obtained by the SSDS are considered high. To the best of our knowledge, no studies report performance on E-town except [18], which used a supervised method. The supervised method reached S=0.957 and $F_{1}=0.856$ in Test 2, compared to the average performance of S=0.951 and $F_{1}=0.871$ by the SSDS. The $F_{1}$ score is higher for the SSDS, but there is a small drop in the S score. Nonetheless, this small drop in the score performance is justified owing to our approach’s practicality and the unrealistic assumption of labeled-attack availability in supervised methods.

## 4. Conclusions

The growing implementation of internet of things (IoT) technology in WDSs exposes them to cyber vulnerabilities. Therefore, the timely detection and localization of cyber-attacks are essential to maintaining system reliability. Most previous studies have developed detection systems, but localizing cyber-attacks is still an understudied problem [3]. Furthermore, the available detection algorithms require datasets with labeled attacks that are rarely available in real-world applications. As such, this paper developed a practical detection and localization methodology that does not rely on a predetermined list of labeled attacks. Nonetheless, our method requires a hydraulic understanding of the network topology since it is built around DMAs or pressure zones within the WDS.

The results show that combining machine learning and statistical tools (MCCA, SVDD) with a physical understanding of WDS characteristics can yield a practical and efficient solution for detecting and localizing cyber-attacks. The performance of the SSDS without any parameter tuning is comparable to state-of-the-art methods that rely on datasets with labeled attacks. The performance of the SSDS was also examined under several sensitivity analyses. The results show robustness over a wide range of parameter values and data exposure levels, implying that the method can be applied without long historical records and parameter tuning. Furthermore, the developed attack-localization mechanism shows excellent localization capabilities, where the system misidentified only 1 out of 14 events. In our system, the attacked DMA is identified without specifically referring to the attacked components within the DMA. While identification at the DMA level might be enough for practical applications, we will examine methods for identifying altered sensors in our future research. For example, we hypothesize that it will be possible to perform sub-zoning for the identified DMA to narrow down the attack identification to one or more sensors. 

## Figures and Tables

**Table 1:** Sensor type and count in the C-town case study.

DMA	Level	Flow	Pressure
**#1**	2	3	4
**#2**	1	2	2
**#3**	1	1	2
**#4**	1	1	2
**#5**	2	2	2

**Table 2:** Performances of the SSDS on the test sets in the two case studies.

	*C-Town*		*E-Town*	
*Index*	Test 1	Test 2	Test 1	Test 2
*S*	0.962	0.953	0.970	0.932
*S_CM_*	0.950	0.949	0.965	0.952
*S_TTD_*	0.974	0.956	0.976	0.912
*F*_1_	0.855	0.884	0.860	0.882
*Precision*	0.789	0.830	0.767	0.829
*Recall*	0.933	0.946	0.979	0.942

**Table 3:** C-Town; performance comparison from the Test 2 dataset.

	*S*	*S_CM_*	*S_TTD_*	*F*_1_	Precision	Recall
VAE [10]	NR	0.812	NR	0.651	0.558	0.781
AE [17]	NR	NR	NR	**0.886**	**0.887**	0.885
SSDS	0.953	**0.949**	0.956	0.884	0.830	**0.946**
Multistage Model [7]	**0.955**	0.947	0.963	NR	NR	NR
OCDS [18]	0.954	0.926	**0.981**	NR	NR	NR

Note: NR = Not Reported.

**Figure A1:**
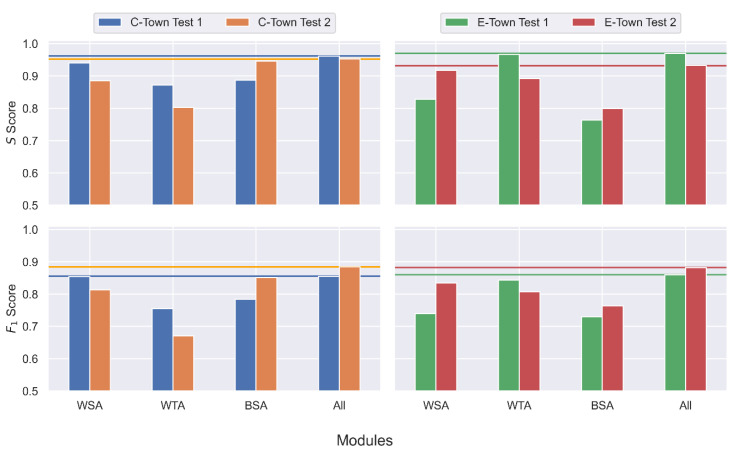
Performance of the SSDS modules. Individual modules (in bars) against all modules combined (in ‘All’ bars and horizontal lines).

**Figure A2:**
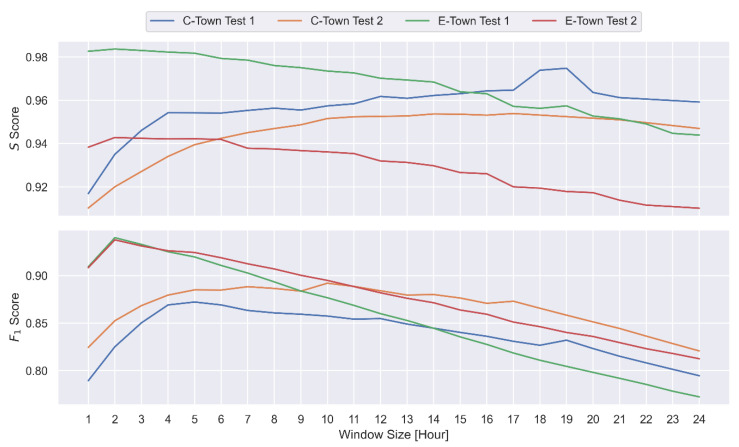
SSDS performance over different window sizes in the smoothing rolling window.

**Figure A3:**
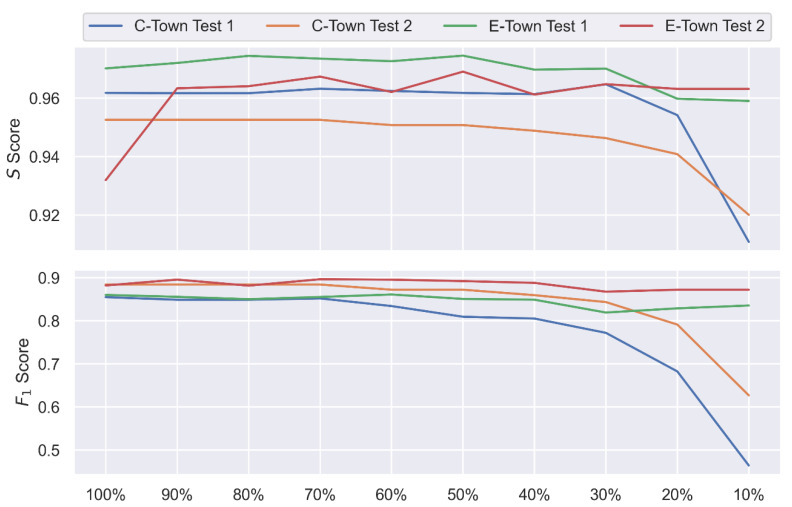
SSDS performance under different levels of data exposure.

## Data Availability

The code and data are provided in GitHub, https://github.com/SWSLAB/SSDS, accessed on 4 August 2022.

## Appendix A. Examining Individual Module Contribution

In the first sensitivity analysis, we examine the contribution of the three modules of the SSDS. In Figure A1, we compare the performance of each module separately to the benchmark of the three modules combined, as suggested in this work. For the C-Town case study, when comparing the individual modules, we found that the module with the lowest performance was the WTA (see the left panels in Figure A1). The low performance of the WTA compared to the other modules can suggest a weak daily pattern in C-Town. It is noted that despite the low performance of the WTA in C-Town, it does not degrade the overall performance when the three modules are combined. The WSA performs best in Test 1, while the BSA module is the best performer in Test 2. This highlights the need to combine the different modules to achieve robust and generalizable results for different datasets.

For the E-Town case study, when comparing the performance of the individual modules, we found different contributions by the modules compared to C-Town. For E-Town’s Test 1, the performances of the WSA and the BSA are inferior (green bars in Figure A1); however, in Test 2 (red bars in Figure A1), the WSA has the best performance among all modules. Unlike in C-Town, the WTA module has the highest scores in Test 1, which implies a strong daily pattern in the E-Town case study. Compared to the low performance in C-Town, this higher performance of the WTA in E-Town further highlights the importance of combining the different modules to reach a good, generalizable performance among different networks and different systems.

## Appendix B. Examining the Impact of Window Size

In the synthesis module (Figure 5), we rely on a retrospective moving average with a predetermined window size that the user can set. In the results section, we used a window size of 12 timesteps for all runs. The logic behind the value of 12 is due to the hourly sampled readings in the case studies, such that we choose to smooth this based on the previous half-day. Since the window size, w, is the only parameter in the suggested algorithm, we performed a sensitivity analysis to demonstrate its effect on the results, as shown in Figure A2. From Figure A2, we can see that the selected window size of 12 is not ideal for any of the datasets in our case studies. Yet, it produces very good results among all datasets. In fact, there is a relatively large domain in which the window size produces satisfactory results compared to other published studies. For example, compared to [17], the SSDS will yield good performance ($F_{1}\geq 0.85$) for any value of window size in the range of 3–13. For the C-Town scores (blue and orange), we can see that the maximum S is obtained by a window size of 19 in Test 1 and 17 in Test 2, while the maximum $F_{1}$ score is obtained by a window size of 5 in Test 1 and 10 in Test 2. Thus, in C-Town, the S score prefers larger window sizes (w=18), while the $F_{1}$ score prefers smaller window sizes (w=7). For the E-Town case study, we see that the performance is generally decreasing with increasing window size. As such, the choice of window size of 12 is far from ideal in the E-Town case study. Still, since our SSDS does not rely on labeled attacks, we could not know these facts in advance, and despite the use of non-ideal window size of w=12, very good results are obtained for both the E-Town and the C-Town case studies.

## Appendix C. Examining the Impact of Data Exposure

The availability of training attack-free samples is not trivial at all. In C-Town and E-Town, we have one year of samples, but in this sensitivity analysis, we tested the performance with different levels of exposure to the training dataset (attack-free dataset) while assuming that only the latest samples were available. For example, a 40% exposure level implies that the last 40% of the training samples were used in offline training. Figure A3 shows that the performance is almost indifferent to the decrease in the exposure level until about a 50% exposure level, after which, C-Town’s performance started to drop (See  at 50%), while in E-Town, the high performance is maintained, even for a lower data exposure of 10% (there is a small drop in E-Town Test 1 after exposure level 30%). These results indicate that the SSDS can perform well at half of the size of the training set (half a year). It is noteworthy that in E-Town’s performance (green and red lines), we can see an enhancement if we drop the first 10% of the samples (exposure level of 90%). This can indicate that the samples at the beginning of E-Town’s training dataset are different from those in Test 1 and Test 2, which results in the inferior performance of the SSDS when these samples are included. This result indicates that the best performance (in terms of S score) of the SSDS can be obtained if we focus on the most recent half a year for training the system. In this case, the SSDS will obtain an S score above 0.95 in all tests. One can argue that taking only the most updated half a year for the training phase is justified since we want to train the system to the most recent normal behavior.
